# The Wild Side of Disease Control at the Wildlife-Livestock-Human Interface: A Review

**DOI:** 10.3389/fvets.2014.00027

**Published:** 2015-01-14

**Authors:** Christian Gortazar, Iratxe Diez-Delgado, Jose Angel Barasona, Joaquin Vicente, Jose De La Fuente, Mariana Boadella

**Affiliations:** ^1^SaBio (Health and Biotechnology), IREC (CSIC – UCLM – JCCM), Ciudad Real, Spain; ^2^Departamento de Sanidad Animal, Facultad de Veterinaria, Universidad Complutense de Madrid, Madrid, Spain; ^3^Department of Veterinary Pathobiology, Center for Veterinary Health Sciences, Oklahoma State University, Stillwater, OK, USA; ^4^SABIOtec Spin-Off, Edificio Polivalente UCLM, Ciudad Real, Spain

**Keywords:** monitoring, population control, shared infections, vaccination, vector control, zoning

## Abstract

The control of diseases shared with wildlife requires the development of strategies that will reduce pathogen transmission between wildlife and both domestic animals and human beings. This review describes and criticizes the options currently applied and attempts to forecast wildlife disease control in the coming decades. Establishing a proper surveillance and monitoring scheme (disease and population wise) is the absolute priority before even making the decision as to whether or not to intervene. Disease control can be achieved by different means, including: (1) preventive actions, (2) arthropod vector control, (3) host population control through random or selective culling, habitat management or reproductive control, and (4) vaccination. The alternative options of zoning or no-action should also be considered, particularly in view of a cost/benefit assessment. Ideally, tools from several fields should be combined in an integrated control strategy. The success of disease control in wildlife depends on many factors, including disease ecology, natural history, and the characteristics of the pathogen, the availability of suitable diagnostic tools, the characteristics of the domestic and wildlife host(s) and vectors, the geographical spread of the problem, the scale of the control effort and stakeholders’ attitudes.

## Introduction

Diseases shared with wildlife are multi-host infections with an impact on human health, economy, and wildlife management or conservation were wildlife itself plays a significant role on infection maintenance. Shared diseases represent a significant burden that affects public health, global economies, and the conservation of biodiversity (1–3). It has been suggested that 80% of the relevant animal pathogens present in the United States of America have a potential wildlife component (4). Furthermore, the number of emerging infectious disease (EID) events caused by pathogens originating in wildlife has increased significantly over time, suggesting that EIDs represent an increasing and highly significant risk to global health (5). Moreover, changes in wildlife management such as changes in harvesting/culling, conservation measures and translocations, feeding and fencing of natural habitat are among the drivers of zoonotic pathogen emergence (6). A collaborative effort of multiple disciplines in a One Health context is crucial if the health of human beings, livestock, wildlife, and the environment is to be improved (7). It is also widely accepted that the total eradication of a shared infectious agent is almost impossible if wildlife hosts, which serve as a natural reservoir of the pathogen are ignored (8–10).

**Figure 1:**
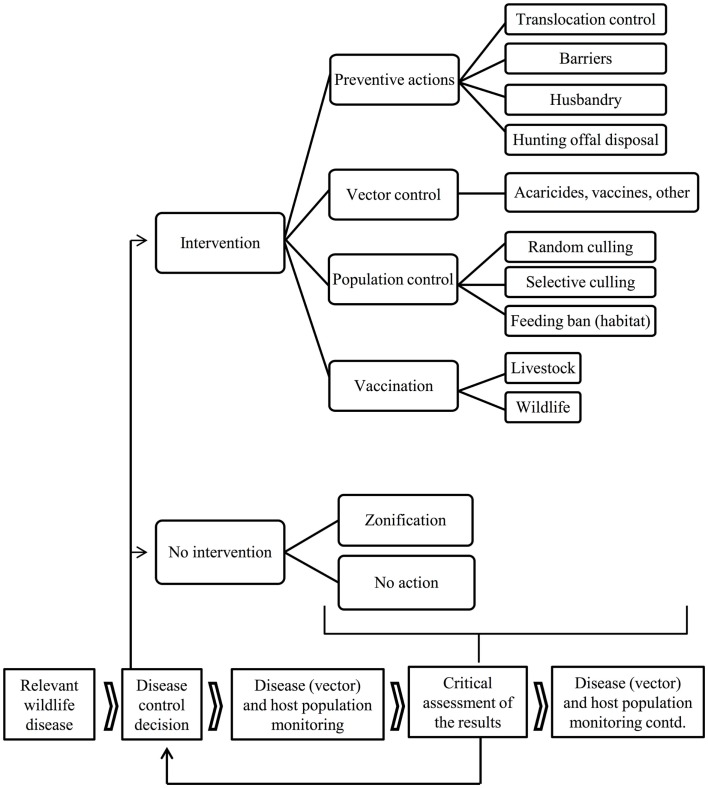
**Flowchart of the available disease control options and result assessment in diseases shared with wildlife**.

**Figure 2:**
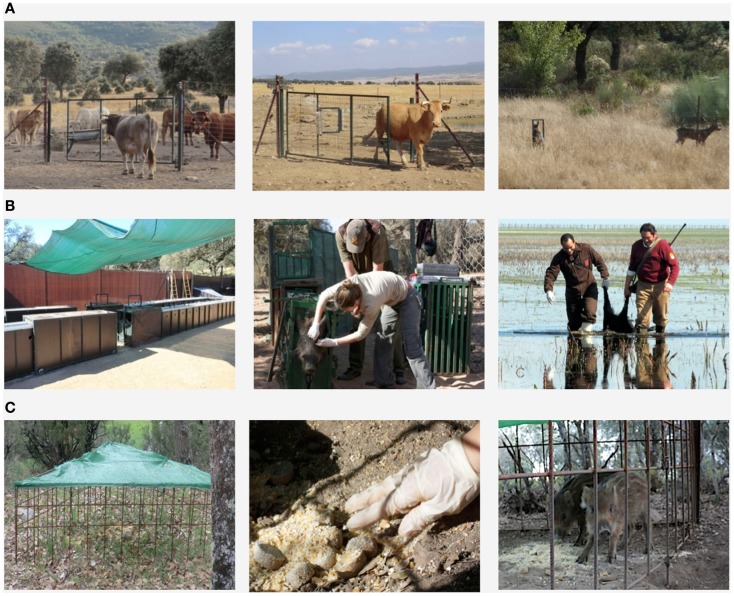
**Examples of some disease control options currently applied: (A) Farm biosecurity by segregating wildlife and cattle using fences [Source: Barasona et al. (42)]; (B) Selective and random culling; (C) Vaccination against TB in wild boar using oral baits**.

Disease emergence in wildlife (e.g., chronic wasting disease, CWD), and difficulties in the eradication of endemic shared diseases such as classical swine fever (CSF) and tuberculosis (TB), have, over the last few decades, prompted a growing interest in disease control in wildlife reservoirs (4, 11–14). The control of diseases shared by wildlife requires the development of strategies to reduce pathogen transmission between wildlife and domestic animals or human beings. The control of wildlife disease often consists of an intervention in natural ecosystems and is, as such, often controversial (14). This review describes the options that are available for disease control at the wildlife-livestock-human interface, from preventive measures to population control and vaccination. This includes a critical review of the options currently applied and an attempt to forecast wildlife disease control in the coming decades. This review does not include those disease control efforts that are directed solely toward wildlife for conservation or game management purposes. Modeling (if not accompanied by actual intervention) is also beyond the scope of this paper. An outline if the steps and options that could be used to achieve disease control are shown in Figure 1 and some examples can be seen in Figure 2.

## Disease Monitoring in Wildlife

The key requisite for any disease control in wildlife is that of establishing a proper surveillance and monitoring scheme. Surveillance and monitoring build on the steady collection, collation, and analysis of data related to animal health but differs at the aim and target population. Surveillance targets wildlife populations classified as healthy to demonstrate the absence of infection (15). Conversely, monitoring focuses on known infected wildlife populations aiming to detect spatial and temporal trends (16). Disease control measures are only undertaken when disease is present; therefore, from now on this paper will focus on monitoring (since surveillance is applied when infection is absent). After disease discovery, descriptive studies are needed in order to assess whether the disease and the role of wildlife is relevant for public or animal health or for wildlife conservation and management. If this is the case, then wildlife diseases must be monitored by defining the key wildlife hosts, host population background data and samples; choosing the appropriate methods for diagnosis and for space-time trend analysis, and establishing a reasonable sampling effort with suitable sample stratification (17). Each situation must be analyzed independently since being a “reservoir” or “spillover host” depends not only on the pathogen and wildlife species but other factors, e.g., wild boar in the Iberian Peninsula are considered reservoir hosts for *M. bovis* but feral hogs in Australia are considered spill over hosts [see more examples in Ref. (18)]. If properly performed, monitoring will allow changes in disease occurrence to be identified and the impact of any intervention to be critically assessed [e.g., Ref. (19)]. One example of the current trend as regards improved wildlife disease monitoring is the European research consortium APHAEA, whose goal is to harmonize approaches in order to develop a health surveillance network for wildlife at a European level by improving both population and disease monitoring (20).

## Disease Control Options

The primary means to control diseases shared by wildlife include (1) preventive actions, (2) arthropod vector control (if vector-borne), (3) host population control through random or selective culling, habitat management or reproductive control, and (4) vaccination. Ideally, tools from several fields should be combined in an integrated control strategy. Targeted and effective methods aiming to maintain natural environments will receive most support despite being potentially controversial (21). Alternative options such as zoning [sensu (14)] or no-action should also be considered, particularly in view of a cost/benefit assessment (Figure 1), but disease and population monitoring are always required.

## Preventive Actions to Control Diseases

Disease prevention at the wildlife-livestock-human interface is a broad field that includes control methods such as translocation, fencing, feed, and water management, farm biosecurity and hygienic hunting-offal disposal, among others.

### Translocation control

Movement control, known as translocation control in wildlife, is one of the most fundamental preventive actions in disease control for both domestic animals and wildlife (22–24). Translocation control is meant to prevent the introduction or re-introduction of pathogens via the release of infected free-living or captive wildlife. Global wildlife trade affects millions of individuals annually, with severe implications for disease emergence (25). Several recent reviews discuss the importance of translocation control for disease prevention [e.g., Ref. (26, 27)], and new regulations have been enforced in some countries (e.g., OIE regulations for chytrid fungus control in amphibians, Royal Decree 1082/2009 in Spain).

### Barriers

This concept includes the use of large or small-scale fencing and any other barrier: physical, dogs, deterrents, barriers to vectors, etc. to prevent the transmission of diseases between animal populations by decreasing contact among them.

#### Large-scale fencing

Certain livestock diseases, such as foot-and-mouth disease (FMD), are difficult to control due to the large numbers of infected wildlife hosts. This limits the ability to trade livestock products in international markets. Fencing has been used on very large scales to segregate wildlife from cattle. One successful example is from southern Africa where livestock and game-proof fences lengthier than 500 km where set up to prevent the spread of rinderpest and FMD (28, 29). However, fences are vulnerable to certain animal species [e.g., suids may slip under them, or elephants may destroy them (30)] being difficult and expensive to maintain. Expenses and doubts on efficacy are some of the reasons why EU Commissioners did not back up proposal of Lithuania Minister on building a fence along Belarus’ border to prevent wild boar movement in order to control African Swine Fever (ASF) spread (31, 32). Moreover, fencing may be an important impediment to conservation as such large barriers seriously interfere with animal migration (33).

#### Farm-biosecurity, small-scale fencing, and deterrents

Although on a far smaller scale, fencing is a key tool in farm biosecurity. Farm biosecurity is becoming a prominent method to prevent infectious disease transmission and reduce wildlife-livestock interactions (34, 35). For example, industrial pig and poultry farms maintain their low disease status partly because they are effectively separated from potentially infected wildlife by fencing and other physical barriers. Farm-biosecurity continues to be improved, not only in intensive rearing facilities but also in open air systems and in livestock production systems in which wildlife contact is likely in pastures, water points or feed-storage sites. On UK cattle farms, appropriately deployed simple exclusion measures (sheet metal gates and fencing, feed bins and electric fencing) were 100% effective in preventing the Eurasian badger (*Meles meles*) from entering farm buildings. These exclusion measures also reduced the level of badger visits to the rest of the farmyard, thus potentially decreasing the risk of *Mycobacterium bovis* (TB) transmission between badgers and cattle (35). Wild ungulates, including white-tailed deer (*Odocoileus virginianus*) and Eurasian wild boar (*Sus scrofa*), are among the most damage-causing wildlife species. Fencing has been demonstrated to reduce the use of potential contact sites by wildlife [e.g., Ref. (36–38)]. In Riding Mountain N.P. (Canada) local fencing was combined with the use of guard dogs to further decrease the risk of *M. bovis* transmission to cattle in conjunction with the on-going test and cull and a deer feeding ban to reduce the risk of elk (*Cervus elaphus*) and white-tailed deer transmitting *M. bovis* to cattle (39). Segregating wildlife and livestock from common resources such as waterholes or feed by setting up selective enclosures or by training dogs to reduce wildlife visits to farms may prove beneficial (40, 41). Actions to prevent disease transmission at water points and feeding sites may also include dispersing or modifying the available water points and replacing feeding sites on the ground with selective feeders which are less accessible to certain species. For instance, an apparent reduction of 66% in cattle TB skin reactors was achieved using fencing to segregate waterholes for either cattle or wildlife on a farm in Spain (42). Care must be taken to select the appropriate segregation method; if it is applied incorrectly it can cause the opposite effect. For example, the policy of massively feeding elk during the winter in the Yellowstone Ecosystem (WY) in order to limit transmission of *Brucella abortus* in pastures shared with cattle may actually contribute toward disease transmission and maintenance within elk herds (43).

### Husbandry

Changes in animal husbandry include infinite possibilities as regards dealing with specific biosecurity problems. These changes include timing and the use or certain pastures, feeding livestock inside, or changing disease susceptible livestock species to less risky ones (44). For instance, agencies can promote substituting horses for ruminants or sheep for cattle in TB endemic areas. The latter option is occasionally being recommended to cattle owners in highly prevalent regions with high wildlife densities in Spain (C. Gortazar, personal communication).

### Carcass and hunting-offal disposal

Another important field in biosecurity and wildlife disease control is the proper removal of harvested animals (including viscera and other remains) in order to limit potential infection spread, principally by mammals (45). One specific case is the obligatory pre-movement testing of hunter-harvested wild boar carcasses for CSF. Wild boar shot, in potentially endemic areas, must remain (refrigerated in appropriate set ups to enable carcass maintenance until clearance) at the hunting site until blood and spleen have been analyzed for CSF in the corresponding laboratory [e.g., Ref. (46)]. In New Zealand, similar discussions are occurring around the movement of potentially *M. bovis* infected feral pig heads collected by hunters as trophies (G. Nugent, personal communication).

The disposal of carcasses and hunting remains has significantly contributed to wildlife disease-related conflict between hunters, government agencies, the livestock industry, and conservationists in Mediterranean Spain (47). Recent field tests have revealed that simple and inexpensive fence designs prevent non-target species, including wild boar, from accessing the food provided for endangered avian scavengers [*Gyps fulvus*, *Aegypius monachus*, *Corvus corax*, and *Aquila adalberti* (48)]. More observational and experimental research is needed in all the aforementioned control methods, since only a few of these methods have been scientifically assessed for their actual contribution to disease control.

## Arthropod Vector Control

The control of arthropod vector infestations for the control of diseases shared with wildlife has principally been described in relation to West Nile virus (WNV) and tick-borne infections such as Lyme borreliosis and babesiosis. West Nile exemplifies the complex interactions between health and the environment (49) as new conflicts are surfacing around culicoid mosquitoes control and environmental health (50). Since there are no efficient vaccines or treatments available for WNV, efforts are focused on vector control mainly by using insecticides though new strategies based on symbionts, such as *Wolbachia* sp (51). Nevertheless, there is an increased concern about the toxic effects of insecticides on non-target insect populations, on human beings and the environment [e.g., Clean Water Act versus pesticide use and wetland management practices such as drainage in Sacramento – San Joaquin Bay – Delta estuary, CA, USA (50)].

*Ixodes* tick control (including habitat management through burning, the use of acaricides, and white-tailed deer elimination) has been shown to reduce *Ixodes scapularis* populations by up to 94%, and acaricide application to deer decreased nymphal *I. scapularis* populations by up to 83%. However, the effect of these strategies on the incidence of Lyme disease in human beings remains unknown (52, 53).

Control efforts for *Babesia* sp. vectors rely on culling wild ungulates in infected and neighbor farms in conjunction with acaricide control of tick infestations in the area. The systematic culling of white-tailed deer as a tick eradication method is regarded as unfeasible due to its high cost, regulations preserving wildlife in American Indian reservations and the ethical considerations behind this approach (54). Pasture rotation methods to reduce the tick burden initiated in the 1970s appear to have failed due to the abundance of white-tailed deer and other wild ungulate species (55, 56).

Two other methods to control ticks on white-tailed deer exist: acaricides and vaccination. Acaricides include systemic treatments through the consumption of ivermectin-medicated corn and/or topical treatments using 4-poster deer treatment bait stations and/or 2-poster deer treatment feeder adapters, both of which passively apply acaricide topically to deer (55). Vaccines against cattle ticks became available in the early 1990s as a cost-effective alternative for tick control that reduced acaricide use as well as the associated problems such as the selection of acaricide-resistant ticks, environmental contamination and the contamination of milk and meat products with acaricide residues (57, 58). Vaccination trials with commercial vaccines containing the *Rhipicephalus microplus* BM86 and BM95 gut antigens, Gavac^®^ and TickGARD^®^ (Heber Biotec S. A., Havana, Cuba and Hoeschst Animal Health, Australia), reduced the number of engorging female ticks, their weight and their reproductive capacity, thus resulting in the reduction of tick infestations and in the prevalence of some tick-borne pathogens (57, 58). Other candidate protective antigens such as subolesin (SUB) have recently been proposed for the control of different tick species and other ectoparasites (59). Vaccination with BM86 and SUB tick protective antigens have reduced tick infestations in red deer (*Cervus elaphus*) and white-tailed deer with an overall vaccine efficacy of approximately 80% for the control of *R. microplus* infestations in white-tailed deer (60).

## Wildlife Population Control

Many factors contribute to the natural regulation of wildlife abundance. Herbivores, which are likely to be particularly relevant for shared disease maintenance, are probably limited by food availability and predation or hunting harvests (61). Disease itself is a mechanism that may regulate wildlife populations. The problem of overabundant wildlife populations and thus, an increased reservoir population, may occasionally be addressed by using relatively simple management actions such as feeding bans or increased harvesting (24, 39, 62).

It has been demonstrated that the supplementary feeding of red deer has a strong effect on the reproductive success of hinds, and hence on population productivity (63). However, feeding bans will have little to no effect on overabundant populations that are not provisioned, such as those in protected areas [e.g., Ref. (64)]. Feeding bans have been known to generate conflict with hunters and landowners if baiting and feeding is perceived as a traditional and rewarding practice by which to increase the hunting harvest (24, 65) or other perceived values (e.g., deer as a symbol of natural resources for Michiganders (66).

The total elimination of a reservoir species is impractical, expensive, and ethically and ecologically unacceptable unless it targets an introduced species (67). Moreover, hunting has limitations in its ability to control wildlife populations, for example, in protected areas or urban habitats, and the effects of culling are only temporary if population control is not sustained over time. It is also known that eliminating or substantially reducing the number of abundant species can have indirect effects on other species. For instance, fox numbers increased after badger culling for TB control in the UK (68); and deer and moose (*Alces alces*) numbers increased, as well as grazing pressure and habitat damage, when carnivore culling was conducted in Canada (69). Culling also has effects over the targeted species such as increased movement due to social disruption [dispersal and immigration; (70–73)] and compensatory reproduction (74). The aforementioned reasons have led some authors to state that culling reservoir populations in order to mitigate or control the transmission of pathogens has proven disappointingly inefficient (14, 75–77) and EFSA to advise against the wild boar mass culling carried out to control ASF transmission in some EU member estates (78).

Random culling may be considered for overabundant populations of introduced species or game species if feeding bans and sustainable habitat management are not feasible. Random culling to control overabundance should be explored before testing other more costly means. As shown in Table 1, random culling can, under certain circumstances, contribute to wildlife disease control. Models suggest that in pathogens that depend on frequency-dependent transmission, culling or increased harvesting can eradicate the disease when birth or recruitment induces the compensatory growth of new, healthy individuals, which has the net effect of reducing disease prevalence by dilution (79). Harrison et al. (80) proposed that the use of wildlife culls for disease control should be proposed only when: (i) the pathogen transmission cycle is fully understood including all the host (vector) interactions; (ii) the response of wildlife populations to culling is known; and (iii) a cost-benefit analysis shows that increased revenue or benefit from reduced disease prevalence exceeds the cost of culling. In practice, random culling is seldom a stand-alone tool but rather one of several elements of an integrated disease control strategy, often based on vaccination.

**Table 1:** **Attempts to control diseases shared with wildlife through population control**.

Type of population control	Wildlife species; pathogen targeted; site	% Population reduction achieved; % infection reduction in wildlife	Efficacy (in terms of reduced contact or infection in livestock or human beings)	Reference
Culling and hazing (bison outside the park are hazed back or culled)	Bison; *Brucella abortus*; Yellowstone, Montana, USA	Negligible; n.a.	Cattle incidents continue	(81)
Random culling	Wild boar; *Mycobacterium bovis*; Spain	50%; 21–48%	Wild boar abundance correlated with annual% of skin test reactor cattle; TB lesion prevalence declined in sympatric red deer	(62)
Random culling	Wild boar; *Mycobacterium bovis*; Spain	67%; Negligible	TB lesion prevalence declined in sympatric fallow deer	(82)
Random culling (local proactive culling)	Badger; *M. bovis*; RBCT, UK	69–73%; n.a.	Variable. Greater effects on cattle breakdowns during post-culling period	(73, 83)
Random culling (widespread proactive culling)	Badger; *M. bovis*; Ireland *(*four areas)	n.a.: 25%	52–82% less of confirmed cattle restrictions	(84)
Random culling (reactive culling)	Badger; *M. bovis*; Laois Co., Ireland	n.a.: n.a.	Higher survival time to future bTB episodes in cattle herds	(85)
Random culling (den gassing)	Badger; *M. bovis*; Avon, UK	n.a.: n.a.	Substantially reduced risk of infection for cattle and no new cases in 10 years	(86, 87)
Random culling	Red deer and wild boar; *M. bovis*; Brotonne, France	Close to 100% in red deer and significant in wild boar; 86%, 82%	No new cattle breakdowns since 2006	(88)
Random culling	Possum; *M. bovis*; New Zealand	Locally close to 100%; n.a.	92% decline in number of infected herds	(39)
Random culling	Feral water buffalo; *M. bovis*; Australia	99%; 100%	100%	(89)
Random culling (restricted + restricted feeding and baiting)	White-tailed deer; *M. bovis*; Michigan, USA	n.a.: 63% but still maintenance hosts	Herd breakdowns continue	(90)
Random culling (intense + feeding and baiting ban)	White-tailed deer; *M. bovis*; Minnesota, USA	50%; 100%	Minnesota regained TB free status in 2010	(24, 90)
Random culling	European starling; *Salmonella enterica*; Texas, USA (feedlots)	66%; n.a.	No apparent reduction in cattle, but disappeared from feed bunks and substantially declined within water troughs	(91)
Random culling	White-tailed deer; Ticks (*Borrelia burgdorferi* vectors); Moneghan island, Maine, USA	100%; Significant tick abundance reduction	n.a.	(92)
Random culling	Wild boar; CSF virus; French Vosges Forest, France	Hunting biased to piglets and juveniles; negligible	No measurable effect	(93) and references therein
Random culling	Wild boar; Suid Herpesvirus 1 – Aujeszky’s disease virus; Spain	50%; 0%	n.a. (no pigs present on treatment sites)	(62)
Random culling (several studies)	Fox and other carnivores; Rabies virus; Europe and North America	Variable; not sufficient	n.a.	(67) and references therein
Selective culling	Bison (fenced wood bison); *B. abortus*; Elk Island NP, Canada	n.a.: 100%	n.a. (no cattle present on treatment site)	(94)
Selective culling (+vaccination of calves)	Elk and Bison (fenced plains bison); *B. abortus*; Elk Island NP, Canada	n.a.: 100%	n.a. (no cattle present on treatment site)	(94)
Selective culling	African buffalo; *M. bovis*; Kwazulu/Natal, South Africa	n.a.: 50%	n.a. (no cattle present on treatment site)	(95)
Selective culling	White-tailed deer; *M. bovis*; Michigan, USA	Negligible; 0%	n.a.	(96)
Selective culling	White-tailed deer; Chronic Wasting Disease (prion); Colorado, USA	Negligible; estimated to take 5–10 years to reduce from 8% to <2%	Locally feasible, but not in large areas owing to costs ($300/animal plus personnel time)	(97)

*n.a., not available*.

A more socially acceptable alternative to random culling is selective (or targeted) culling, similar to test and cull schemes applied to domestic animals. Such actions can be very expensive, and their feasibility depends on access to the animals, the availability of convenient, sensitive and specific tests, the prevalence of the infection, and the spatial distribution of the target population (Table 1).

Random and selective culling strategies are more likely to succeed in isolated populations than on large geographical scales, and the results will probably consist of a certain reduction of disease prevalence in the wildlife host and in the domestic host targeted, rather than in the total eradication of the infectious agent (94). The success of a culling scheme will also depend on the attributes of the specific infectious agent targeted (62). Increased research into random and selective culling, with simultaneous alternative methods such as immunocontraception or feeding bans, is needed. Indeed, fertility control methods as immunocontraception are perceived by the general public as a more acceptable manner for limiting wildlife population than culling (98, 99). Immunocontraception may as well be a tool to control venereal and vertical transmitted diseases (100) and has several advantages over culling as no compensatory reproduction or behavior disturbances take place (101). However, long-term effectiveness and side effects have to be further investigated (102).

## Vaccination and Medication

In this context, wildlife vaccination to reduce infection prevalence emerges as a valuable alternative or complementary tool in disease control. Disease control through the vaccination of wildlife reservoirs may potentially have advantages over other approaches. As opposed to culling, vaccination may be more acceptable to the general public (103) since it is a non-destructive and sustainable (does not increase the susceptible animals in the population) method of controlling disease in wildlife.

The best vaccination method for wildlife populations spread over a wide geographical area is oral vaccination using baits. The oral vaccination of wildlife is the only disease management tool with proven efficacy on large spatial scales. This has been shown most clearly in the case of fox rabies control in Western Europe (104). Table 2 summarizes the most significant wildlife vaccination assays carried out in the field, and their outcomes. Many more host/pathogen binomia are currently being evaluated in the laboratory or are beginning to be investigated in preliminary field studies [e.g., Ref. (103)]. Such on-going studies are not included in this review.

**Table 2:** **Attempts to control diseases shared with wildlife through vaccination**.

Pathogen targeted; Wildlife species; Site	Vaccine deployment	% Reduced infection in wildlife	Reference
Classical Swine Fever virus; Wild boar; France	Oral (preventive vaccination)	n.a., Effective prevention of infection maintenance	(105)
Foot-and-Mouth Disease virus; Buffalo and other wildlife; South Africa	Cattle vaccination in contact areas with infected wildlife	n.a., Breakdowns linked with fence permeability, vaccination coverage, and efficiency of animal movement control measures	(106)
*Mycobacterium bovis*; Badger; UK	Parenteral	61–72% Reduction in the incidence of positive test results	(107)
*M. bovis*; Possum; New Zealand	Oral	95–96%	(108)
Rabies virus; Coyote; Texas, USA	Oral	100%	(109)
Rabies virus; Gray fox; Texas, USA	Oral	n.a.	(109)
Rabies virus; Raccoon; Ontario, Canada	Oral	n.a., Contributed to geographical containment	(110)
Rabies virus; Raccoon; Wolfe Island, Ontario, Canada	Oral and parenteral (+rabies-caused mortality)	100%	(111)
Rabies virus; Red fox; Germany	Oral	100%	(104)
Rabies virus; Red fox; Ontario, Canada	Oral	Close to 100%, but persists in skunks	(110, 112)
Rabies virus; Red fox; and raccoon dog Estonia	Oral	100%	(113)

*n.a., not available*.

However, wildlife disease control can eventually interfere with wildlife ecology. In diseases where vaccination significantly reduces target host mortality, effects on sympatric prey, predators or competitors may occur (110, 114) while this is unlikely for chronic and endemic diseases. In addition, some management tools commonly used to improve bait deployment, such as artificial feeding, are known drivers of reproductive success (63) and can increase wildlife spatial aggregation at feeding sites (115). As discussed previously, these methods can actually increase disease transmission if applied on a wide scale for prolonged periods of time. Vaccines must demonstrate biosafety for non-target species [vaccines against diseases, such as CSF, that affect only one species do not represent a risk for non-target species; (105)] and physical stability to endure environmental temperature conditions, though inactivated vaccines circumvent this requirement [some effective oral inactivated vaccines are already being developed, (116)]. Approaches within natural ecosystems should therefore first be carefully tested in trials that are progressively extended to a larger scale (14).

Medication of wild animals can rarely be used to reduce the burden of disease in wild populations and very few examples exist in the literature of the medication of free-ranging wildlife in comparison to the plethora of reports on vaccination. Among these, the control of *Echinococcus multilocularis* in foxes is a prominent example. The adult fox tapeworm is sub-microscopic and infects foxes and, less efficiently, dogs. The larval form infects several wild rodents. In villages and small towns in central Europe, foxes are responsible for environmental *E. multilocularis* egg contamination in the vicinity of human beings, leading to infection risk if human beings accidentally ingest viable eggs (117). The knowledge developed for fox rabies vaccine delivery through oral baits has been built on to employ similar strategies by which to deploy the anthelminthic praziquantel (118).

An important concern when releasing drugs into the environment is biosafety (119, 120). Though, the presence of anthelmintic compounds in the environment is mainly derived from their massive use in the livestock industry.

## Compartmentalization and Zoning: Knowing the Problem and Living with it

Both compartmentalization and zoning (or zonification) can and have been implemented by countries or states in order to define sub-populations of varying health statuses for disease control. This could become one of the best solutions for disease control at the wildlife-livestock interface in the future [see Ref. (14) for a recent review]. The idea of zoning consists of defining a geographical area in which an infection exists in order to differentiate its infection status from other zones. This has, for example, been proposed for Yellowstone bison (*Bison bison*), suggesting that the inherent cost of declaring a brucellosis-infected zone would be far lower than current management to avoid *Brucella abortus* spillback to cattle (121). It is also carried out *de facto* as regards *M. bovis* and *B. abortus* infected wood bison in Wood Buffalo N.P. in Alberta, Canada (39, 94) and for several wildlife species carrying FMD in Namibia and Zimbawue (28, 29).

A related concept is compartmentalization, during which segregation is based on production-linked establishments and types of animal husbandry and biosecurity, rather than on geographical boundaries. Free-ranging domestic pigs could, for instance, belong to a different (and more at risk) compartment than industrial pigs, thus allowing a different status to be defined for each compartment.

## Economic Effects of No Action

Inaction is a frequent decision in the control of wildlife diseases. This is due to the fact that, for most diseases, there is no strong justification for intervention (in terms of public or animal health conservation) or if justification exists there are no suitable and cost-efficient disease control tools available (12). Regardless, the decision to take no action should be accompanied by monitoring in order to assess the effect of this inaction on pathogen maintenance and on animal and human health. This would allow our strategy to be changed if monitoring proves that our decision should be reconsidered (12).

Taking no action to control diseases can result in higher costs. One example is the dramatic increase in prevalence of TB in badgers after the suspension of TB cattle testing during the FMD epidemic in the UK in 2000–2001. This was ascribed to the high prevalence of cattle herd infection and cattle with advanced disease (70). In New Zealand, the control of the invasive Australian brushtail possum (*Trichosurus vulpecula*) ceased during an economic crisis in the early 1980s. Almost immediately, cattle TB prevalence rose (P. Livingstone, personal communication). Modeling offers a useful alternative approach to the development of management criteria and facilitates the consideration of ecological-economic trade-offs, signifying that diseases are managed in a cost-effective manner (122, 123).

## Wildlife Disease Control in the 21st Century: Toward Integrated Disease Control Schemes

Various general inferences can be made from the review given above. First, setting up a proper disease and population surveillance and monitoring scheme is an absolute priority; even before deciding whether or not to intervene (Figure 1). For example, the information provided by the European research consortia APHAEA and ANTIGONE constitutes valuable knowledge with which to start up a surveillance network (20, 124). Second, all options for disease control at the wildlife-livestock-human interface, including those of no intervention, need to be considered, either individually or combined. Third, combining several disease control tools in integrated strategies is likely to reduce the cost and effort required for disease control. Integrated strategies are also preferred since no single control measure is universally applicable (125). However, when more than one tool is used in a control strategy, the relative contribution of each one is confounded (90, 111). Fourth, the success of disease control in wildlife depends on many factors, including (a) the single or multi-host nature and other characteristics of the pathogen, (b) the availability of suitable diagnostic tools, (c) the characteristics of the wildlife host(s) and vectors, (d) the geographical range of the pathogen/reservoir (improved control in isolated versus continuous populations) and the scale of the control effort (large-scale longitudinal programs are better), (e) the attitude of the stakeholders involved (highly dependent on their education and communication provided to them).

One particular field deserving increased attention is the One Health approach, meaning a need for better collaboration between public health, veterinary, and environment services in order to address shared diseases. For instance, game species depend on veterinary services while on the farm, on environment services after their release into the wild, and on public health services after being harvested for human consumption. Despite this fact, inter-agency information exchange and collaboration is often limited. To overcome this difficulty, governments should consider setting up “One Health working groups,” aimed at improving inter-agency collaboration for instance through specific information exchange mechanisms and through joint risk assessment exercises considering not just one of the three compartments [e.g., Ref. (126)]. Also, the potential of wildlife rescue centers for the monitoring and early detection of potentially zoonotic or economically relevant diseases is often neglected (127). In fact, disease in wildlife populations has been compared to an iceberg with only the tip of the total mass being visible at any time (12) because there were few people looking for it and other considerations related to the wilderness of wildlife (difficulties in detecting and measuring disease and individuals their selves). Nowadays, several surveillance and monitoring schemes are operating in wildlife worldwide (128–131) and generating a considerable amount of valuable information. As mentioned earlier, the number of EID events caused by pathogens originating in wildlife and the risk they represent to global health evidences the necessity of engagement between these wildlife specialists and other agencies (WHO, OIE).

Most current monitoring and disease control efforts in wildlife are directed toward only a few relevant diseases, including rabies, ASF, CSF, FMD, CWD, brucellosis, TB, *E. multilocularis*, and tick-borne diseases. In the future, it is likely that this list will become longer as new scenarios and disease control needs emerge. Future wildlife disease control efforts will probably rely on a better understanding and modeling of wildlife–pathogen interactions (123), thus improving biosafety and prevention. Other fields expected to grow include immunocontraception for population control, selective culling and, most notably, vaccination. New vaccines will hopefully permit more cost-effective, biosafe, and cheaper disease control in wildlife. Recent results with inactivated *M. bovis* vaccines (116, 132) and recombinant arthropod vector vaccines for the control of both vector infestations and pathogen transmission (59, 133) support this research direction. The development of effective vaccines for wildlife is still in its infancy, but the results reviewed here have demonstrated the possibilities and advantages of integrated control strategies, and encourage support to expand research in this area in order to contribute to the eradication of wildlife-associated diseases.

Finally, from a global point of view, disease control schemes should be aimed at the accomplishment of a balance. Most of the above-mentioned examples of shared wildlife diseases are resultant of unbalanced situations in which, for instance, wildlife has increased in numbers, often as the result of anthropogenic factors [such as rural abandonment or land use changes (2)]. Any proposed control scheme that does not target re-establishing an ecological balance will probably be limited to a short-term success instead of long-term disease control.

## Conflict of Interest Statement

The authors declare that the research was conducted in the absence of any commercial or financial relationships that could be construed as a potential conflict of interest.
